# Psychosocial impact of testing human papillomavirus positive in Australia's human papillomavirus‐based cervical screening program: A cross‐sectional survey

**DOI:** 10.1002/pon.5897

**Published:** 2022-02-12

**Authors:** Verity Chadwick, Kirsty F. Bennett, Kirsten J. McCaffery, Julia M. L. Brotherton, Rachael H. Dodd

**Affiliations:** ^1^ Royal North Shore Hospital St Leonards New South Wales Australia; ^2^ Department of Behavioural Science and Health Cancer Communication and Screening Group University College London London UK; ^3^ Faculty of Medicine and Health School of Public Health The University of Sydney Sydney New South Wales Australia; ^4^ VCS Population Health VCS Foundation Carlton South Victoria Australia; ^5^ Melbourne School of Population and Global Health University of Melbourne Carlton Victoria Australia

**Keywords:** anxiety, cancer, early detection of cancer, oncology, papillomavirus infections, psychological distress

## Abstract

**Objective:**

To examine the impact of self‐reported human papillomavirus (HPV) test result (HPV negative, HPV positive, HPV result unknown) on a range of psychosocial outcomes.

**Methods:**

Women and other people with a cervix in Australia aged 25–74 years who reported having participated in cervical screening since December 2017 were recruited through Facebook and Instagram to complete an online survey. The primary outcome measures were anxiety, emotional distress, and general distress.

**Results:**

Nine hundred fifteen participants completed the online survey; 73.2% reported testing HPV negative (‘HPV−’), 15% reported testing HPV positive (‘HPV+’) and 11.8% reported that they did not know/remember their test result (‘HPV unknown’). Compared to participants testing HPV−, participants testing HPV+ had higher mean anxiety (41.67 vs. 37.08, *p* < 0.001) and emotional distress scores (11.88 vs. 7.71, *p* < 0.001). Concern about test result (34.3% vs. 1.3%, *p* < 0.001), perceived risk compared to average women (55.4% vs. 14.1%, *p* < 0.001), and cancer worry (27.8% vs. 5.9%, *p* < 0.001) were also greater among HPV+ participants than participants testing HPV−. Participants testing HPV+ felt less reassured about their screening result than participants testing HPV− (16% vs. 75.1%, *p* < 0.001). Participants testing HPV+ had greater knowledge of HPV (11.96 vs. 10.36 out of 16, *p* < 0.001) and HPV testing (3.94 vs 3.28 out of 5, *p* < 0.001) than participants who reported testing HPV−.

**Conclusions:**

Elevated levels of anxiety and emotional distress were found in those testing HPV+ compared with those testing HPV−. Future research should examine what strategies should be used to deliver test results and what additional information is provided, in order to alleviate anxiety among individuals testing HPV+.

## Key points

- Anxiety and emotional distress are significantly greater in individuals who report testing human papillomavirus positive (HPV+)
- Knowledge of human papillomavirus (HPV) is high in individuals who report testing HPV+, but there are still some significant knowledge gaps
- Future research is needed to examine how individuals should best receive HPV test results, and what resources should be provided

## 1 INTRODUCTION

Cervical cancer is the fourth most commonly diagnosed cancer worldwide,^1^ but in Australia cervical cancer prevalence is at an all‐time low.^2^ This is largely due to high uptake of cervical screening and widespread uptake of the human papillomavirus (HPV) vaccine.^3^ The HPV‐based screening program, introduced in December 2017, is thought to be more effective at reducing incidence and mortality due to cervical cancer than the previous program using cervical cytology (Pap) testing.^4^ HPV testing has higher sensitivity for the detection of pre‐cancerous lesions, facilitating longer screening intervals.^4^

Given that HPV is transmitted sexually, prior research has shown shame and stigma to be associated with the disclosure of positive results to partner, family and friends; judgment from others; and the belief that negative connotations (such as sexual promiscuity) are associated with HPV.^5^ We conducted a survey in 2017 to measure anxiety and distress levels shortly following the implementation of HPV‐based screening, and found participants (women and other people with a cervix) who received HPV+ results, had significantly higher levels of anxiety and distress.^6^ These effects are similar to responses observed in the broader sexually transmitted infection (STI) literature.^7^ Fears of malignancy, partner infidelity, potential transmission, pain caused by colposcopy and treatment, and of future infertility have commonly been reported post communication of a HPV+ result.^5^ These psychosocial stressors may have become more widespread now that HPV testing is a routine part of cervical screening, as around 8% of women and other people with a cervix test HPV+ in Australia's program.^2^ Although only a small proportion of these individuals may experience anxiety or distress due to a HPV+ result, given the large numbers who attend for cervical screening each year, this would still equate to a large number of individuals experiencing a heightened emotional response.^2^ Given the potential proportion of individuals testing HPV+ since implementation of the renewed cervical screening program, this study aimed to examine the psychosocial impact of primary HPV screening among Australians a further year into the renewal of the cervical screening program, in a sample recruited via social media.

## 2 MATERIALS AND METHODS

### 2.1 Sample

Eligible participants were individuals residing in Australia aged 25–74 who had undergone cervical screening since 1 December 2017 under the renewed National Cervical Screening Program (NCSP). Participants were excluded if they had no capacity to consent, had a previous diagnosis of cervical cancer or had undergone a hysterectomy.

### 2.2 Procedure

Participants were recruited using targeted paid advertising on Facebook and Instagram between 13 and 23 March 2020, to ‘complete a short survey about cervical screening’. Participants could enter a prize draw to win one of twenty $AUD20 vouchers. A web‐link directed participants to read the participant information statement, provide consent to participate (via a tick‐box), and complete a cross‐sectional survey eliciting sociodemographic, clinical, and other background information.

### 2.3 Measures

Participants completed sociodemographic and clinical information adapted from previous studies of cervical screening attitudes in women/patients.^8^, ^9^ Primary outcomes were anxiety, measured using the State‐Trait Anxiety Inventory (STAI‐6),^10^ and emotional and general distress, using the Cervical Screening Questionnaire^11^ and General Health Questionnaire (GHQ‐12)^12^ respectively. Secondary outcomes included psychosexual distress (modified versions of the Psychosocial Effects of Abnormal Pap Smears Questionnaire short‐form [PEAPS‐Q‐5]),^13^ cancer worry, understanding of HPV results,^14^ perceived risk of developing cervical cancer,^15^ concern regarding screening results,^16^ future screening intention^17^ and a validated measure of knowledge of HPV (16 items) and HPV testing (5 items).^18^

#### 2.3.1 Perceived risk of cervical cancer

Perceived lifetime risk was assessed using ‘What are your chances of developing cervical cancer in your lifetime?’ (no/low/medium/high chance; recoded as ‘no/low chance’ and ‘medium/high chance’).^15^ Perceived risk compared to another woman with similar characteristics was assessed using ‘What is your lifetime chance of getting cervical cancer compared to a woman of your age and race without any known risk factors?’ (much below/slightly below average/same average risk recoded as ‘average/below average’; slightly above/much above average recoded as ‘above average’).^19^

#### 2.3.2 Cancer worry

Worry about developing cervical cancer was assessed using ‘How worried are you of getting cervical cancer in your lifetime?’ (not at all/a bit worried recoded as ‘lower worry’; quite/very worried recoded as ‘higher worry’).^15^

#### 2.3.3 Concern about test results

Concern about test results was assessed using ‘How concerned do you feel about your recent cervical screening result?’ (not at all/slightly/somewhat concerned recoded as ‘lower concern’; moderately/very concerned recoded as ‘higher concern’).^16^

#### 2.3.4 Reassurance about test results

Reassurance about test results was assessed using ‘How reassured do you feel about your recent cervical screening result?’ (not at all/slightly reassured recoded as ‘lower reassurance’; somewhat/moderately/very reassured recoded as ‘higher reassurance’).^16^

#### 2.3.5 Knowledge of HPV and HPV testing

Knowledge of HPV was assessed using ‘Before today had you ever heard of human papillomavirus (HPV)?’ (yes/no). Those responding ‘yes’ answered statements assessing knowledge of HPV (16 statements) and HPV testing (5 statements) as ‘true’, ‘false’ or ‘don't know’).^18^ All ‘don't know’ responses were recoded as ‘incorrect’ and a total knowledge score was calculated for knowledge of HPV (range 0–16) and HPV testing (range 0–5).

### 2.4 Sample size

With a minimum sample of 1000 participants, and assuming approximately 7.5% of the sample is HPV+ and equal standard deviations of 14.6 on the STAI in both HPV+ and HPV− groups (based on previous work),^6^ a 95% confidence interval no wider than ±3.44 units (0.24 standard deviations) around the mean would be achievable.

### 2.5 Analysis

Analyses were carried out using SPSS v22. Differences in the demographic characteristics between the three result groups (HPV+, HPV−, HPV unknown) were described descriptively. Variation in anxiety, general distress, emotional distress, knowledge of HPV and HPV testing across the three result groups were explored using ANCOVA. In the anxiety, general distress and emotional distress analyses, we adjusted for age, education, country of birth, relationship status, and cervical screening pre‐December 2017. In the knowledge of HPV and HPV testing analyses, we additionally adjusted for HPV vaccine receipt. After establishing main effects, post‐hoc tests using a Bonferroni adjustment were used to explore differences between groups.

Multivariate logistic regression models explored whether perceived risk of cervical cancer, cancer worry, and concern and reassurance about test results were associated with HPV test result outcome (adjusting for age, education, country of birth, relationship status and cervical screening pre‐December 2017). Odds ratios, indicating increased or decreased odds for the outcome for each group compared to the HPV− group, with 95% confidence intervals and *p *values, were calculated.

Data collection was stopped prior to the minimum sample being achieved due to the start of the COVID pandemic, to minimize general anxiety and distress measures being confounded by the pandemic.

## 3 RESULTS

Of the 100,712 impressions (number of advertisement views; views may not be unique each time), 2302 clicked through to the survey. From this, 1312 began the survey and 922 unique responses were completed and eligible for analysis. Participants (*n* = 7) with an ‘unsatisfactory sample’ were excluded from the analysis. Characteristics of the 915 participants are shown in Table 1. Participants had a mean age of 38.5 years (SD = 11.6). Most participants were educated to University/College degree level (90.9%), married or living with a partner (68.9%), and nearly half were in full‐time employment (46.8%). The majority of participants were born in Australia (84.4%), with 1.4% identifying as Aboriginal or Torres Strait Islander. Around half reported having received the HPV vaccine (50.2%). Most participants (91.3%) had received a Pap smear prior to December 2017 and around two‐thirds of women (67%) had been screened recently, in 2019 or 2020, with 51.5% reporting regular cervical screening (every 2 years). Most participants reported testing HPV− at their most recent cervical screening test (73.2%) with 15% testing HPV+ and 11.8% reporting that they did not know/could not remember their screening result.

**TABLE 1:** Sample characteristics (*n* = 915)

	HPV+ (*n* = 137)	HPV− (*n* = 670)	HPV unknown (*n* = 108)	Total (*n* = 915)
	*n* (%)	*n* (%)	*n* (%)	*n* (%)
Age				
25–39	101 (73.7)	412 (61.5)	61 (56.5)	574 (62.7)
40–54	24 (17.5)	157 (23.4)	35 (32.4)	216 (23.6)
55–69	11 (8.0)	97 (14.5)	12 (11.1)	120 (13.1)
70–74	1 (0.7)	4 (0.6)	0 (0)	5 (0.5)
Level of education^a^				
High	124 (90.5)	610 (91.0)	98 (90.7)	832 (90.9)
Medium	7 (5.1)	39 (5.8)	5 (4.6)	51 (5.6)
Low	6 (4.4)	21 (3.1)	5 (4.6)	32 (3.5)
Employment				
Full time	84 (61.3)	297 (44.3)	47 (43.5)	428 (46.8)
Part time	33 (24.1)	221 (33.0)	40 (37.0)	294 (32.1)
Other	20 (14.6)	152 (22.7)	21 (19.4)	193 (21.1)
Relationship status				
Single/dating	44 (32.1)	114 (17.0)	16 (14.8)	174 (19.0)
Married/living with partner	70 (51.1)	481 (71.8)	79 (73.1)	630 (68.9)
Partnered/not living with partner	18 (13.1)	36 (5.4)	4 (3.7)	58 (6.3)
Other	5 (3.6)	39 (5.8)	9 (8.3)	53 (5.8)
Born in Australia				
Yes	116 (84.7)	562 (83.9)	94 (87.0)	772 (84.4)
Aboriginal or Torres Strait Islander				
Yes	‐	9 (1.6)	2 (2.1)	11 (1.4)
Non‐English‐speaking background				
Yes	‐	14 (2.1)	‐	14 (1.5)
Received HPV vaccine				
Yes	79 (57.7)	333 (49.7)	47 (43.5)	459 (50.2)
No	56 (40.9)	317 (47.3)	55 (50.9)	428 (46.8)
Don't know	2 (1.5)	20 (3.0)	6 (5.6)	28 (3.1)
Doses of HPV vaccine received (*n* = 460)				
1	2 (2.5)	9 (2.7)	2 (4.3)	13 (2.8)
2	9 (11.4)	45 (13.5)	3 (6.4)	57 (12.4)
3	46 (58.2)	225 (67.6)	31 (66.0)	302 (65.8)
Don't know	22 (27.8)	54 (16.2)	11 (23.4)	87 (19.0)
Pap smear prior to Dec 2017				
Yes	125 (91.2)	611 (91.2)	99 (91.7)	835 (91.3)
No/don't know	12 (8.8)	59 (8.8)	9 (8.3)	80 (8.7)
Frequency of Pap smears				
More frequently than recommended <2 years	36 (28.8)	83 (13.6)	14 (14.1)	133 (15.9)
Regular – every 2 years	63 (50.4)	328 (53.7)	39 (39.4)	430 (51.5)
Less frequently than recommended >2 years	22 (17.6)	160 (26.1)	39 (39.4)	221 (26.5)
Only one	4 (3.2)	40 (6.5)	7 (7.1)	51 (6.1)
Year of last cervical screen				
2017	1 (0.7)	43 (6.4)	8 (7.4)	52 (5.7)
2018	10 (7.3)	219 (32.7)	21 (19.4)	250 (27.3)
2019	91 (66.4)	333 (49.7)	50 (46.3)	474 (51.8)
2020	35 (25.5)	75 (11.2)	29 (26.9)	139 (15.2)

^a^ Education split into high (university degree, diploma or certificate), medium (trade apprenticeship or higher school certificate) and low (school certificate or less or no school/other qualification). Population data for education from 2016 census (high: postgraduate diploma, graduate diploma, bachelor degree, advanced diploma, certificate 3 and 4, medium: year 10 and above, certificate 1 and 2, and low: year 9 or below and no educational attainment).

### 3.1 General anxiety and distress

Mean scores for general anxiety, general distress, and emotional distress by HPV status are presented in Table 2. Anxiety differed significantly across the three HPV result groups (*F*[2,899] = 5.97, *p* = 0.003) and was highest among the HPV+ group (*X̅* = 41.67, SE = 4.05) and lowest among the HPV− ( *x̅* = 37.08, SE = 3.88) and HPV unknown groups ( *x̅* = 37.79, SE = 4.04). Post‐hoc tests revealed that participants in the HPV+ group had significantly higher anxiety scores than participants in the HPV− group (*p* = 0.002). There were no differences between the HPV+ and HPV unknown group (*p* = 0.098) or the HPV− and HPV unknown groups (*p* = 1.00).

**TABLE 2:** Adjusted mean scores for emotional‐ and knowledge‐related outcomes by HPV status

	Possible range of scores	Observed range of scores	Mean scores by HPV status (SE)			Mean scores by HPV status (SE)					*p* value for pairwise comparisons	
			Unadjusted			Adjusted						
			HPV+	HPV−	HPV status unknown	HPV+	HPV−	HPV status unknown	*p*	HPV+ versus HPV‐	HPV+ vs. HPV unknown	HPV− vs. HPV unknown
Emotional^a^												
Anxiety (STAI‐6)	20–80	20–80	45.74 (1.18)	40.52 (0.53)	41.17 (1.33)	41.67 (4.05)	37.08 (3.88)	37.79 (4.04)	0.003	0.002	0.098	1.00
General distress (GHQ‐12)	0–12	0–12	3.36 (0.30)	2.75 (0.14)	2.91 (0.34)	3.35 (1.02)	2.95 (0.98)	3.10 (1.02)	0.475	0.691	1.00	1.00
Emotional distress (CSQ)	0–27	0–26	12.29 (0.28)	8.00 (0.13)	8.35 (0.31)	11.88 (0.94)	7.71 (0.90)	8.01 (0.94)	<0.001	<0.001	<0.001	1.00
Psychosexual distress (PEAPS‐Q)^b^	1–6	1–5	1.99 (0.90)			2.30 (0.32)						
Knowledge^c^												
HPV Knowledge	0–16	0–16	13.36 (0.27)	11.50 (0.12)	10.69 (0.30)	11.96 (0.94)	10.36 (0.90)	9.66 (0.93)	<0.001	<0.001	<0.001	0.091
HPV Testing knowledge	0–5	0–5	4.03 (0.12)	3.27 (0.06)	2.96 (0.14)	3.94 (0.40)	3.28 (0.39)	3.00 (0.40)	<0.001	<0.001	<0.001	0.195

^a^ Adjusted for age, education, country of birth, relationship status and cervical screening pre‐December 2017.

^b^ Only women in the HPV+ group were asked to complete the PEAPS‐Q items.

^c^ Adjusted for age, education, country of birth, relationship status, cervical screening pre‐December 2017 and HPV vaccine receipt.

General distress, measured by the GHQ‐12, did not differ across the three HPV result groups (*F*[2, 899] = 0.75, *p* = 0.475). Post‐hoc tests also revealed no significant differences between the HPV+ and HPV− groups (*p* = 0.691), HPV+ and HPV unknown groups (*p* = 1.00) or the HPV− and HPV unknown groups (*p* = 1.00).

### 3.2 Cervical screening specific emotional distress

Emotional distress, measured by the CSQ, was significantly different across the three HPV result groups (*F*[2,899] = 92.70, *p* < 0.001) and was highest among the HPV+ group ( *x̅* = 11.88, SE = 0.94) and lowest among the HPV− ( *x̅* = 7.71, SE = 0.90) and HPV unknown groups ( *x̅* = 8.01, SE = 0.94). Post‐hoc tests revealed that participants who reported testing HPV+ had significantly higher emotional distress scores than participants who reported testing HPV− and HPV unknown (both *p* < 0.001). There was no difference between the HPV− and HPV unknown groups (*p* = 1.00).

### 3.3 Perceived risk

Compared to participants who reported testing HPV−, participants who reported testing HPV+ were significantly more likely to perceive themselves to be at above average risk of developing cervical cancer in their lifetime (56.9% vs. 20.9%, OR = 4.69, 95% CI: 3.14–6.99, *p* < 0.001; Table 3) and of developing cervical cancer compared to another woman with similar characteristics (55.4% vs. 14.1% OR = 7.3, 95% CI: 4.8–11.1) *p* < 0.001). Participants who did not know their HPV status were also more likely to perceive themselves to be at above average risk of developing cervical cancer compared with those testing HPV− (30.6% vs. 20.9%, OR = 1.63, 95% CI: 1.03–2.57, *p* = 0.037).

**TABLE 3:** Odds of reporting psychosocial outcomes by HPV result group (compared to HPV− group)

	Proportion reporting each psychosocial outcome			Unadjusted odds of reporting psychosocial outcome		Adjusted odds of reporting psychosocial outcome	
	HPV−	HPV+	HPV status unknown	HPV+	HPV status unknown	HPV+	HPV status unknown
	*n* (%)	*n* (%)	*n* (%)	OR (95% CI)	OR (95% CI)	OR (95% CI)	OR (95% CI)
Perceived risk in lifetime^a^	140 (20.9)	78 (56.9)	33 (30.6)	5.01 (3.40–7.36)***	1.67 (1.06–2.61)*	4.69 (3.14–6.99)***	1.63 (1.03–2.57)*
Perceived risk compared to average women^b^	95 (14.1)	76 (55.4)	22 (20.4)	7.54 (5.05–11.26)***	1.55 (0.92–2.59)	7.31 (4.82–11.11)***	1.53 (0.91–2.58)
Cancer worry^c^	40 (5.9)	38 (27.8)	6 (5.6)	6.05 (3.70–9.89)***	0.93 (0.38–2.24)	5.81 (3.47–9.73)***	0.89 (0.36–2.17)
Concern about test result^d^	9 (1.3)	47 (34.3)	4 (3.7)	38.35 (18.18–80.90)***	2.83 (0.85–9.34)	38.90 (17.77–85.15)***	2.63 (0.79–8.81)
Reassurance about test result^e^	503 (75.1)	22 (16.0)	45 (41.7)	0.13 (0.09–0.19)***	0.27 (0.17–0.43)***	0.13 (0.09–0.20)***	0.26 (0.17–0.42)***

*Note*: Adjusted for age, education, country of birth, relationship status and cervical screening pre‐December 2017.

^a^ Women responding ‘medium chance’ or ‘high chance’ to the item ‘What are your chances of developing cervical cancer in your lifetime?’.

^b^ Women responding ‘slightly above average’ or ‘much above average’ to the item ‘What is your lifetime chance of getting cervical cancer compared to a woman of your age and race without any known risk factors?’.

^c^ Women responding ‘quite worried’ or ‘very worried’ to the item ‘How worried are you of getting cervical cancer in your lifetime?’.

^d^ Women responding ‘moderately concerned’ or ‘very concerned’ to the item ‘How concerned do you feel about your recent cervical screening result?’.

^e^ Women responding ‘moderately reassured’ or ‘very reassured’ to the item ‘How reassured do you feel about your recent cervical screening result?’.

*p < 0.05, **p < 0.01, ***p < 0.001.

### 3.4 Cancer worry

Compared to participants who reported testing HPV−, participants who reported testing HPV+ had increased odds of reporting high worry about developing cervical cancer (27.8% vs. 5.9%, OR = 5.81 95% CI: 3.47–9.73) *p* < 0.001). There was no difference between the HPV− and HPV unknown group (*p* = 0.688).

### 3.5 Concern and reassurance about test results

Compared to participants who reported testing HPV−, participants who reported testing HPV+ had increased odds of reporting high concern about their test results (34.3% vs. 1.3%, OR = 38.9, 95% CI: 17.77–85.15, *p* < 0.001). There was no difference between the HPV− and HPV unknown group (*p* = 0.117). Participants who reported testing HPV+ had lower odds of reporting reassurance about their test results (16% vs. 75.1%, OR = 0.13, 95% CI: 0.09–0.20, *p* < 0.001), as did participants who did not know their HPV status (41.7% vs. 75.1%, OR = 0.26, 95% CI: 0.17–0.42, *p* < 0.001).

### 3.6 Knowledge of HPV and HPV testing

The percentage of correct responses ranged from 29% for the item ‘HPV usually doesn't need any treatment’ to 96.4% for the item ‘HPV can cause cervical cancer’ (Appendix 1). Most participants knew that HPV can be passed on during sexual intercourse (89.7%), but only 48.4% knew that most sexually active people will get HPV at some point in their lives. While 88.6% of participants correctly identified the statement ‘If a woman tests positive for HPV she will definitely get cervical cancer’ as being false, only 57.5% knew that if a HPV test shows that a woman does not have HPV that their risk of cervical cancer is low.

Knowledge of HPV and HPV testing differed significantly across the three HPV result groups (knowledge of HPV: F[2, 883] = 18.68, *p* < 0.001; knowledge of HPV testing: (F[2, 804] = 16.24, *p* < 0.001; Table 2). Knowledge was highest in the HPV+ group (knowledge of HPV: *X̅* = 11.96, SE = 0.94; knowledge of HPV testing: *X̅* = 3.94, SE = 0.40) and lowest in the HPV unknown group (knowledge of HPV: *X̅* = 9.66, SE = 0.93; knowledge of HPV testing: *X̅* = 3.00, SE = 0.40). Post‐hoc tests revealed that participants who reported testing HPV+ had significantly greater knowledge of HPV and HPV testing than participants who reported testing HPV− and HPV unknown (all *p* < 0.001). There were no significant differences in knowledge of HPV between the HPV− and HPV unknown groups (*p* = 0.091), or knowledge of HPV testing between these groups (*p* = 0.195).

## 4 DISCUSSION

This study found higher levels of anxiety and emotional distress in those testing HPV+ compared with those testing HPV− in the context of the Australian cervical screening program in a highly educated sample. Individuals who reported testing HPV+ were significantly more anxious than those who reported testing HPV−, on both the general anxiety measure (STAI) and the screening specific measure of emotional distress (CSQ). Elevated anxiety for participants who reported testing HPV+ relative to those reporting testing HPV− is consistent with earlier research immediately following the screening changes in 2017,^6^ which showed a difference in mean anxiety using the STAI tool of around 10 points between participants who reported testing HPV+ and participants who reported testing HPV−. Such elevated anxiety levels were thought to be disproportionate to risk; similar levels had been reported in individuals who reported testing HPV+ with abnormal cytology^20^ or individuals about to have a colposcopy.^21^ Indeed, the results of this study, which collected its data just over 2 years following the renewal of the cervical screening program, showed that the difference in mean anxiety between people who reported testing HPV+ and people who reported testing HPV− is around five points.

Although the levels of anxiety are lower among participants who reported testing HPV+ in this sample compared with a previous study,^6^ this study suggests that HPV testing may continue to have an adverse psychological impact on individuals who report testing HPV+ in primary screening beyond 6 month.^22^ This is contrary to previous findings,^22^ as one third of respondents in this sample had their last cervical screening test over 18 months prior to completing the survey. Average anxiety scores among participants who reported testing HPV+ in this sample were still raised well above scores of the general population, consistent with other studies of individuals who reported testing HPV+,^5^ and similar to scores of individuals with other physical signs of STIs.^23^ Qualitative research has found that individuals testing HPV+ report feeling low levels of control, confusion about the meaning of results, concern about developing cancer, and shame regarding the mode of acquiring HPV,^5^ all contributing to emotional distress regarding HPV+ results. It is unclear what information individuals receive with their test results about HPV and the implications of their results, and this requires further investigation.

The lower levels of anxiety demonstrated in this sample among participants who reported testing HPV+ compared to a previous Australian sample^6^ may be driven by improved knowledge about HPV and a longer average interval since the screening test. In comparison to our previous study,^6^ a larger proportion of this sample responded correctly to knowledge questions about HPV and HPV testing. However, sizeable gaps in knowledge were still apparent, with less than half of the respondents aware that most sexually active individuals have HPV,^24^ and only 29% aware that HPV often doesn't require treatment, knowledge known to ameliorate stigma and self‐blame associated with testing HPV+.^5^ These findings support earlier research supporting the need for practitioners to routinely provide tailored information resources about HPV to patients, including possible test results, what they mean, and for those who test HPV+, ongoing management options.^5^, ^25^ Future research is needed to investigate the association between length of time since screening and psychological impact, similar to studies conducted in England.^26^, ^27^

In contrast to anxiety measures, distress specific to cervical screening was higher relative to previous studies.^6^, ^20^ These results might reflect recruitment methodology; Instagram and Facebook users were younger women and may have a higher propensity to use the Internet to search for information about their results. The use of Internet searches for health information has been associated with increased health‐related distress.^28^

Finally, 12% of participants did not know their HPV result from their latest cervical screening test. This proportion is unchanged from a previous Australian sample in 2017 and remains concerning.^6^ It is possible that these participants had forgotten, as some research suggests that results with less emotion attached, that is negative results, become less memorable.^29^ Another possibility is that these participants were not informed of their result or did not understand what they were told. All practices should ensure they have routine procedures in place to inform patients of their results even if they are normal, as anxiety and emotional distress levels in this group were high. Beyond test results, only half of respondents reported attending regularly for cervical screens. This is a lower proportion relative to the 2017 data,^6^ which may be attributable to their younger age, although it is in line with government data that suggests around 52% of Australian women attended regularly for cervical screening when the two yearly interval was in place.^2^

### 4.1 Study limitations

Our sample is self‐selected and differs significantly from the Australian population, with an increased proportion of participants reporting testing HPV+ in comparison to the general public (15% vs. 6.5% in all Australian women),^2^ participants being more educated (90% Bachelor level vs. 37.1% of all Australian women)^30^ and less ethnically diverse (85% Australian born vs. 70% 2016 census).^30^ Nonetheless, our results support prior studies finding that testing HPV+ may be associated with short term psychosocial distress. This survey was conducted as the COVID‐19 pandemic began, when general anxiety may have been somewhat elevated across all participants. However, data collection was stopped early to help minimise the impact of the pandemic on the results. Due to this, we did not reach the required sample size so all results should be interpreted with caution. Also, the differences in anxiety levels between participants who reported testing HPV+ and participants who reported testing HPV− were broadly consistent with the literature. We did not obtain medical data for the participants in this sample and therefore cannot verify their self‐reported cervical screening test result; however, previous studies have shown that respondents are often accurate in reporting their cancer screening history.^31^

### 4.2 Clinical implications

This study highlights elevated anxiety and emotional distress in individuals testing HPV+, two years following the renewal of the NCSP. Understanding what information individuals receive both prior to and following their cervical screening test is pertinent to ensure information needs are addressed in ways which may also minimise psychosocial impact.

### 4.3 Conclusions

Individuals who reported testing HPV+ 2 years following implementation of the renewed NCSP in Australia still reported significantly higher anxiety, concern, and specific distress about test results than those individuals who reported testing HPV−. Future research evaluating the provision of information about HPV, test results and next steps on anxiety and distress, would be valuable.

## CONFLICT OF INTEREST

Julia M. L. Brotherton's employer VCS Foundation has received donated human papillomavirus (HPV) tests kits and equipment for unrestricted research purposes from HPV test manufacturers including Roche, Cepheid, Seegene and Becton Dickinson.

## ETHICS APPROVAL

This study was approved by The University of Sydney Human Ethics Committee (2018/836).

## AUTHOR CONTRIBUTIONS

Rachael H. Dodd and Kirsten J. McCaffery conceived, designed and developed the methods for the study. Rachael H. Dodd coordinated the running of the study. Kirsty F. Bennett and Rachael H. Dodd contributed to the analysis. Verity Chadwick and Rachael H. Dodd drafted the manuscript. Julia M. L. Brotherton contributed to the content and critical analysis. All authors contributed to the interpretation of the analysis and critically revised the manuscript.

## Supporting information

Supporting Information S1

## Data Availability

The data that support the findings of this study are available from the corresponding author, Rachael H. Dodd, upon reasonable request.
