# Supplementary material for: Psychosocial impact of testing human papillomavirus positive in Australia's human papillomavirus‐based cervical screening program: A cross‐sectional survey
Source: Psychooncology. 2022 Feb 12;31(7):1110–9. doi: 10.1002/pon.5897 (PMC9546150; doi:10.1002/pon.5897)
Supplement: Supplementary file 1 — Supporting Information S1 [file PON-31-1110-s001.docx]

# **Appendix 1: The percentage of correct and incorrect responses to individual HPV knowledge and HPV testing items**

|  | Correct  n (%) | Incorrect n (%) |
| --- | --- | --- |
| **HPV knowledge (n=901)** |  |  |
| HPV can cause cervical cancer | 869 (96.4) | 32 (3.6) |
| A person could have HPV for many years without knowing it | 830 (92.1) | 71 (7.9) |
| HPV can be passed on during sexual intercourse | 808 (89.7) | 93 (10.3) |
| HPV always has visible signs or symptoms (F) | 794 (88.1) | 107 (11.9) |
| Using condoms reduces the risk of getting HPV | 766 (85.0) | 135 (15.0) |
| Men cannot get HPV (F) | 751 (83.4) | 150 (16.6) |
| HPV can be passed on by genital skin-to-skin contact | 733 (81.4) | 168 (18.6) |
| Having many sexual partners increases the risk of getting HPV | 714 (79.2) | 187 (20.8) |
| HPV can cause HIV/AIDS (F) | 693 (76.9) | 208 (23.1) |
| HPV is very rare (F) | 683 (75.8) | 218 (24.2) |
| There are many types of HPV | 647 (71.8) | 254 (28.2) |
| HPV can cause genital warts | 604 (67.0) | 297 (33.0) |
| HPV can be cured with antibiotics (F) | 586 (65.0) | 315 (35.0) |
| Most sexually active people will get HPV at some point in their lives | 436 (48.4) | 465 (51.6) |
| Having sex at an early age increases the risk of getting HPV | 353 (39.2) | 548 (60.8) |
| HPV usually doesn’t need any treatment | 261 (29.0) | 640 (71.0) |
| **HPV testing knowledge (n=822)** |  |  |
| If a woman tests positive for HPV she will definitely get cervical cancer (F) | 728 (88.6) | 94 (11.4) |
| When you have an HPV test, you get the results the same day (F) | 575 (70.0) | 247 (30.0) |
| HPV testing is used to indicate if the HPV vaccine is needed (F) | 561 (68.2) | 261 (31.8) |
| If an HPV test shows that a woman does not have HPV her risk of cervical cancer is low | 473 (57.5) | 349 (42.5) |
| An HPV test can tell how long you have had an HPV infection (F) | 420 (51.1) | 402 (48.9) |
